# Childhood maltreatment and adult blood pressure-based outcomes: age- and sex-specific associations in the German National Cohort (NAKO)

**DOI:** 10.1186/s12889-026-29019-z

**Published:** 2026-09-09

**Authors:** Tara Alissa Thielcke, Johanna Klinger-König, Sylvia Stracke, Klaus Berger, Stefanie Castell, Karin Halina Greiser, Volker Harth, Margit Heier, André Karch, Thomas Keil, Lilian Krist, Michael Leitzmann, Seraina Lerch, Marcello Ricardo Paulista Markus, Claudia Meinke-Franze, Rafael Mikolajczyk, Ilais Moreno Velásquez, Nadia Obi, Tobias Pischon, Carsten Oliver Schmidt, Sabine Schipf, Ulrike Siewert-Markus, Henry Völzke, Margarethe Woeckel, Hans J. Grabe, Till Ittermann, Philipp Töpfer

**Affiliations:** 1 Department of Study of Health in Pomerania/Clinical-Epidemiological Research, Institute for Community Medicine, University Medicine Greifswald, Greifswald, Germany; 2 Department of Psychiatry and Psychotherapy, University Medicine Greifswald, Greifswald, Germany; 3 Department of Internal Medicine A, University Medicine Greifswald, Greifswald, Germany; 4 Institute of Epidemiology and Social Medicine, University of Münster, Münster, Germany; 5 Department of Epidemiology, Helmholtz Centre for Infection Research (HZI), Braunschweig, Germany; 6 German Cancer Research Centre (DKFZ) Heidelberg, Division of Cancer Epidemiology, Heidelberg, Germany; 7 Institute for Occupational and Maritime Medicine (ZfAM), University Medical Center Hamburg-Eppendorf, Hamburg, Germany; 8 KORA Study Centre, University Hospital of Augsburg, Augsburg, Germany; 9 Institute of Social Medicine, Epidemiology and Health Economics, Charité - Universitätsmedizin Berlin, Berlin, Germany; 10 Institute of Clinical Epidemiology and Biometry, University of Würzburg, Würzburg, Germany; 11 State Institute of Health I, Bavarian Health and Food Safety Authority, Erlangen, Germany; 12 Institute of Epidemiology and Preventive Medicine, University of Regensburg, Regensburg, Germany; 13 Department of Medical Psychology, University Medicine Greifswald, Greifswald, Germany; 14 German Center for Child and Adolescent Health (DZKJ), partner site Greifswald/Rostock, Greifswald, Germany; 15 Department of Internal Medicine B, University Medicine Greifswald, Greifswald, Germany; 16 German Centre for Cardiovascular Research (DZHK), Partner Site Greifswald, Greifswald, Germany; 17 German Center for Diabetes Research (DZD), Partner Site Greifswald, Greifswald, Germany; 18 Institute of Community Medicine, University Medicine Greifswald, Greifswald, Germany; 19 Institute for Medical Epidemiology, Biometrics, and Informatics (IMEBI), Interdisciplinary Center for Health Sciences, Medical Faculty of the Martin-Luther-University Halle-Wittenberg, Halle, Germany; 20 Max Delbrück Center for Molecular Medicine in the Helmholtz Association (MDC), Molecular Epidemiology Research Group, Berlin, Germany; 21 Max Delbrück Center for Molecular Medicine in the Helmholtz Association (MDC), Biobank Technology Platform, Berlin, Germany; 22 Charité - Universitätsmedizin Berlin, corporate member of Freie Universität Berlin and Humboldt-Universität zu Berlin, Berlin, Germany; 23 Institute of Epidemiology, Helmholtz Zentrum München - German Research Center for Environmental Health, Neuherberg, Germany; 24 Department of Psychiatry and Psychotherapy, LMU University Hospital, LMU Munich, Munich, Germany; 25 German Center for Neurodegenerative Diseases (DZNE), Site Rostock/Greifswald, Greifswald, Germany

**Keywords:** Child maltreatment, Risk factors, Blood pressure, Cardiometabolic risk factors, Sex factors, Age factors

## Abstract

**Background:**

Childhood maltreatment (CM) is associated with adult cardiovascular disease (CVD) risk. Systolic (SBP) and diastolic (DBP) blood pressure (BP), key markers of CVD risk, exhibit age- and sex-dependent variability, which was insufficiently accounted for in previous studies on the CM-BP relationship. This study therefore aimed to assess age- and sex-specific associations between CM and adult BP-based outcomes and hypertension using cross-sectional data from the population based German National Cohort (NAKO).

**Methods:**

Complete data were available for 150,983 participants (49.3% female), who took part in the baseline assessment between 2014 and 2019. CM was assessed via the Childhood Trauma Screener (CTS). Resting BP was assessed using a standardized procedure. Hypertension was defined as either SBP ≥ 140 mmHg or DBP ≥ 90 mmHg or intake of antihypertensive medication. Age- and sex-stratified multivariable regression models estimated age- and sex-specific associations between CM and BP-based markers and sex-stratified logistic regression models were calculated for hypertension as the outcome.

**Results:**

A pattern of inverse associations between CTS sum score (CTS_SUM_) and BP markers emerged. Compared to their non-exposed counterparts, females (OR = 0.87, 95%-CI: 0.80; 0.94) but not males (OR = 1.01, 95%-CI: 0.91; 1.13) with high cumulative CM exposure (i.e., ≥ 3 CM categories) had lower odds of hypertension. Age-dependent inverse associations between CTS_SUM_ and BP markers revealed sex-specific patterns. The age- and sex-dependent inverse associations of CM with BP markers were more consistent for the abuse vs. neglect subscales.

**Conclusions:**

The association between CM and BP revealed age- and sex-dependent patterns, highlighting the complexity of physical consequences in adulthood associated with CM.

**Supplementary Information:**

The online version contains supplementary material available at https://doi.org/10.1186/s12889-026-29019-z.

## Introduction

Exposure to childhood maltreatment (CM) is highly prevalent. Approximately 23% of the population in high-income countries have experienced at least one type of CM [1]. Thus, CM poses a substantial public health burden [2]. Recent research suggests that preventing just 10% of CM cases in Europe and North America could reduce societal costs by approximately $105 billion annually [1].

A major contributor to the disease burden associated with CM is its association with a range of chronic physical and mental health problems in adulthood [1, 3] as well as premature mortality, particularly due to cardiovascular disease (CVD) [4]. Exposure to CM has been shown to increase the risk of ischemic heart disease [5] and cerebrovascular disease, particularly among females [6]. These associations have been attributed to both biological alterations and changes in health-related behaviors [7].

Therefore, associations between CM and common adult CVD risk factors have received considerable attention. For example, previous studies demonstrated higher risks for elevated inflammatory markers [8], diabetes mellitus [3], obesity, smoking and physical inactivity [9] in individuals exposed to CM. However, studies examining the association between CM and elevated adult blood pressure (BP) and hypertension revealed inconsistent results [10, 11], with positive, inverse, and null associations reported for both systolic BP (SBP) [10, 12–17] and diastolic BP (DBP) [13–16]. For instance, a recent meta-analysis reported an 19% increased risk of adult hypertension in people with CM-exposure, but no association with SBP and a very small effect (Cohen´s d = 0.04) on DBP [11]. One possible reason for this inconsistency may be that BP levels vary substantially over the life course in a sex-specific manner [18]. Therefore, results on CM-BP associations may systematically vary as a function of basic sociodemographic sample characteristics i.e., age and sex. This issue has not been adequately addressed in most previous studies, which have instead either adjusted for age and sex, or focused on just one sex [10, 17] or one age group [12]. However, other studies have shown that age [13, 14] and sex [15] may moderate the association between CM exposure and adult BP levels. For example, Souama et al. [14] reported in a Dutch longitudinal study that CM exposure was associated with lower SBP at baseline, but with higher SBP after 9 years of follow-up, without examining potential sex differences. In contrast, Schreier et al. [15] found that CM exposure was associated with lower resting BP, but only among females.

In summary, the existing literature on CM-related BP levels in adulthood remains inconclusive, which may partly be explained by inadequate consideration of age-dependent sex differences. Therefore, we aimed to investigate the age- and sex-dependent associations between CM and BP markers, i.e., SBP, DBP, pulse pressure (PP), in a large, population-based sample from the German National Cohort (NAKO).

## Methods

### Study population

The present study is based on baseline data from the NAKO, a population-based cohort study within Germany aiming to investigate the development of major chronic diseases. Data were acquired between 2014 and 2019 at 18 study centers located in both urban and rural areas across 13 of the 16 German federal states. Participants were randomly selected from local residents’ registration offices using age- and sex-stratified sampling. Approximately 10,000 participants of each sex in the 20–29 and 30–39 age groups, and approximately 27,000 participants of each sex in the 40–49, 50–59 and 60–69 age groups were included, resulting in a total sample of 204,744 subjects [19]. For sequential exclusion due to missing data, see Figure S1.

### Childhood maltreatment

CM was assessed via retrospective self-report using the CTS, a short version of the Childhood Trauma Questionnaire (CTQ). The CTS assesses severity of exposure to forms of abuse (emotional, physical, sexual) and neglect (emotional, physical). For the CTS, one item per CTQ-subscale was selected based on its discriminatory power, explained variation, and practicability [20]. Each item is rated on a 5-point Likert scale, resulting in a CTS_SUM_ of 5–25 points, with higher scores indicating higher severity of exposure to CM. In addition, for dichotomous classification, validated cut-off scores were applied for each CTS subscale individually to indicate moderate-to-severe CM exposure (i.e., ≥ 3 points for emotional and physical abuse, ≥ 4 points for emotional and physical neglect, ≥ 2 points for sexual abuse). An index of cumulative CM exposure was computed based on these dichotomized values to capture the number of different moderate/severe CM subtypes each individual experienced (i.e., none, one, two, three or more) [21].

### Blood pressure (BP)

BP was measured as part of the physical examination after five minutes of rest in a sitting position using the right arm and repeated after 2 min. All measurements were performed using the same model of automated oscillometric BP monitor (HEM 705 IT Omron Healthcare GmbH) [22]. As recommended, values of the second BP measurement were used for analysis [22].

### Hypertension

Hypertension was defined as SBP ≥ 140 mmHg or DBP ≥ 90 mmHg [23] measured on site, or as intake of antihypertensive medication. Participants were instructed to bring all medication taken seven days prior to participation to the examination center. Medication was assessed using anatomical therapeutic chemical (ATC) codes and categorized as antihypertensive medication for ATC codes C02AC, C02CA, C02DB, C02DC, C03A, C03B, C03CA, C03CB, C03D, C03E, C07, C08CA, C08DA, C08DB, C08G and C09.

### Sociodemographic information and covariates

Date of birth and sex of participants were obtained from local registration offices and cross-validated with self-reports at the study center. In cases of incomplete or conflicting registry information, self-reported data were used. Sociodemographic information was obtained as part of a standardized computer-assisted interview. Basic demographic variables provided by participants included sex, age, migration background, years of formal education, and equivalent income. Body height (in cm) was assessed using the Stadiometer 274 (seca GmbH & Co. KG, Hamburg, Germany). Body weight was measured to the nearest 0.1 kg using the digital scale integrated into the Medical Body Composition Analyzer 515 (seca GmbH & Co. KG, Hamburg, Germany) [19]. Body mass index (BMI) was calculated as body weight (kg) divided by body height (m) in meters squared. Smoking status was self-reported using a questionnaire during a touchscreen module. Individuals were subsequently categorized as never, former, and current smokers.

### Statistical analyses

For a descriptive overview, the dataset was stratified by sex and a dichotomized CM variable (at least one moderate to severe CM vs. none). We reported continuous data by means and standard deviations and categorical data by absolute numbers and percentages. Based on sex-specific linear regression models, we plotted the course of BP markers and the CTS_SUM_ over age.

We assessed potential non-linear associations between the exposures and outcomes using multivariable fractional polynomial (MFP) models implemented with the mfp command in Stata. Fractional polynomial models of up to degree two were considered, with powers selected from the predefined set conventionally used in the MFP approach (− 2, − 1, −0.5, 0, 0.5, 1, 2, and 3, where 0 represents the logarithmic transformation). Model selection was based on comparisons of model deviance using the closed-test procedure implemented in the mfp algorithm, with simpler linear specifications retained unless a more complex functional form significantly improved model fit. As no substantial deviations from linearity were identified, the final analyses were conducted using linear terms for the exposures.

The CTS_SUM_ was associated with continuous BP markers by sex-stratified linear regression models adjusted for age, years of formal education, BMI, smoking status, antihypertensive medication, and study center. In addition, formal sex-by-CTS interaction terms were tested to complement the a priori sex-stratified analyses. Fractional polynomials were also tested here, but no substantial deviations from a linear relationship were observed. Results are reported as β-coefficients and 95% confidence intervals (CI). Sex-stratified logistic regression models adjusted for age, years of formal education, BMI, smoking status, and study center were used to associate the CTS_SUM_ with hypertension. Results are reported as odds ratios (OR) and 95% CI’s. In sensitivity analyses, we repeated these models for the cumulative number of moderate/severe CM subtypes experienced by each individual (1 vs. 0; 2 vs. 0; 3 or more vs. 0). Furthermore, CTS subscales were individually associated with the BP markers by sex-stratified multivariable linear and logistic regression models.

Potential effect modification by age was tested via CTS_SUM_-age-interactions on the BP markers in female and male participants, separately. We then calculated multivariable sex-specific regression models in the age groups 20–29 years, 30–39 years, 40–49 years, 50–59 years, and 60 + years. Sex- and age-specific associations between CTS_sum_- and respective BP outcomes were plotted. Age-specific associations between CM subdimensions and BP markers were conducted in a similar fashion. We used STATA 18.5 (Stata Corporation, TX, USA) for all analyses.

## Results

### Sample characteristics

Sample characteristics, stratified by sex and CM exposure, are shown in Table 1. A total of 21,294 females (28.6%) and 17,879 males (23.4%) reported exposure to at least one moderate-to-severe CM. Among these individuals, 13,631 females (64.0%) and 12,549 (70.2%) males stated that they had been exposed to one type of CM, 4,343 females (20.4%) and 3,495 (19.5%) males reported exposure to two types of CM, and 3,320 females (15.6%) and 1,835 males (10.3%) reported three or more types of CM. For both females and males reporting at least one type of CM, physical neglect was the most common CM subtype: 34.7% in females and 42.2% in males. The sex-specific patterns of mean SBP, DBP, and PP at different ages are shown in Figure S2 and of the mean CTS_SUM_ in Figure S3.

**Table 1:** Characteristics of the study population stratified by sex and childhood maltreatment (CM)

	Females		Males	
	CM-	CM+	CM-	CM+
N	53,143 (71.4%)	21,294 (28.6%)	58,667 (76.6%)	17,879 (23.4%)
Age in years	48.0 (12.5)	51.2 (11.4)	48.5 (12.5)	52.5 (11.4)
Years of education				
less than 10 years	801 (1.5%)	778 (3.7%)	540 (0.9%)	471 (2.6%)
10 years	22,758 (42.8%)	10,629 (49.9%)	19,669 (33.5%)	7,452 (41.7%)
more than 10 years	29,584 (55.7%)	9,887 (46.4%)	38,458 (65.6%)	9,956 (55.7%)
Equivalent income	2291 (1341)	2123 (1289)	2564 (1610)	2345 (1592)
Migration Status (yes)	6,985 (13.1%)	3,469 (16.3%)	7,527 (12.8%)	3,122 (17.5%)
Smoking status				
Never smokers	28,911 (54.5%)	9,012 (42.4%)	26,785 (45.7%)	6,160 (34.5%)
Former smokers	15,605 (29.4%)	7,395 (34.8%)	20,274 (34.6%)	7,270 (40.7%)
Current smokers	8,550 (16.1%)	4,847 (22.8%)	11,546 (19.7%)	4,414 (24.7%)
Alcohol consumption in g/day	7.14 (11.93)	7.53 (13.67)	13.89 (18.41)	14.75 (21.52)
CTS_SUM_	5.97 (1.18)	10.67 (3.11)	6.17 (1.23)	10.47 (2.49)
Number of types of CM’s				
0	53,143 (100.0%)	0 (0.0%)	58,667 (100.0%)	0 (0.0%)
1	0 (0.0%)	13,631 (64.0%)	0 (0.0%)	12,549 (70.2%)
2	0 (0.0%)	4,343 (20.4%)	0 (0.0%)	3,495 (19.5%)
3 or more	0 (0.0%)	3,320 (15.6%)	0 (0.0%)	1,835 (10.3%)
Emotional neglect	0 (0.0%)	6,255 (29.4%)	0 (0.0%)	5,002 (28.0%)
Physical abuse	0 (0.0%)	5,755 (27.0%)	0 (0.0%)	6,376 (35.7%)
Emotional abuse	0 (0.0%)	7,354 (34.5%)	0 (0.0%)	4,724 (26.4%)
Sexual abuse	0 (0.0%)	7,152 (33.6%)	0 (0.0%)	1,940 (10.9%)
Physical neglect	0 (0.0%)	7,390 (34.7%)	0 (0.0%)	7,553 (42.2%)
BMI in kg/m^2^	25.66 (5.26)	26.46 (5.70)	26.99 (4.35)	27.65 (4.58)
Waist circumference in cm	84.63 (13.08)	87.00 (13.94)	95.85 (12.60)	98.21 (13.04)
Systolic blood pressure in mmHg	123 (16)	124 (17)	132 (15)	133 (16)
Diastolic blood pressure in mmHg	77 (10)	78 (10)	81 (10)	81 (10)
Pulse pressure; mmHg	46.1 (11.1)	46.4 (11.7)	51.2 (10.6)	51.4 (11.4)
Intake of antihypertensive drugs	9,668 (18.4%)	4,596 (21.9%)	13,518 (23.3%)	5,116 (29.0%)
Hypertension (yes)	16,050 (30.2%)	7,301 (34.3%)	25,883 (44.1%)	9,049 (50.6%)
Pulse in bpm	71 (10)	71 (10)	69 (11)	69 (12)
Type 2 diabetes (yes)	2,259 (4.3%)	1,140 (5.4%)	2,925 (5.0%)	1,349 (7.6%)

Data are expressed as mean and standard deviation for continuous data or as absolute numbers and percentages for categorical data

*Abbreviations: BMI* Body Mass Index, *CM* Childhood maltreatment, *CM*- No CM subtype with moderate to severe exposure, CM+ = ≥1 CM subtype with moderate to severe exposure, *CTS* Childhood Trauma Screener

### Sex-specific associations of CTS_SUM_ with SBP, DBP, PP, and hypertension

After adjusting for age, education, BMI, smoking status, antihypertensive medication, and study center, a one-point increase in the CTS_SUM_ was associated with a 0.20 mmHg reduction in SBP, a 0.05 mmHg reduction in DBP, and a 0.15 mmHg reduction in PP in females (Table 2). In males, a one-point increase in the CTS_SUM_ was associated with 0.15 mmHg reduction in SBP, 0.03 mmHg reduction in DBP, and 0.12 mmHg reduction in PP. The OR for hypertension was 0.98 (95% CI = 0.97; 0.99) in females and 0.99 (95% CI = 0.98; 0.99) in males, respectively. Complementary tests indicate significant sex-by- CTS_SUM_ interactions (Table S1) for SBP (*p*<0.001) and PP (< 0.001), but not for DBP (*p* = 0.327) and hypertension (*p* = 0.706). Overall, associations appeared more consistent in females in sex-stratified analyses, with formal evidence of sex interaction for systolic blood pressure and pulse pressure, but not for diastolic blood pressure or hypertension.

**Table 2:** Sex-specific associations of childhood maltreatment (CM) and cumulative CM with blood pressure markers

Outcome	CTS_SUM_ β (95%-CI)	1 vs. no CM β (95%-CI)	2 vs. no CM β (95%-CI)	3 + vs. no CM β (95%-CI)
Systolic blood pressure; mmHg				
Females	-0.20 (-0.24; -0.16)	-0.69 (-0.97; -0.41)	-1.37 (-1.83; -0.91)	-1.78 (-2.30; -1.26)
Males	-0.15 (-0.19; -0.11)	-0.43 (-0.71; -0.15)	-1.18 (-1.68; -0.69)	-0.85 (-1.52; -0.18)
Diastolic blood pressure; mmHg				
Females	-0.05 (-0.07; -0.03)	-0.12 (-0.29; 0.06)	-0.42 (-0.71; -0.14)	-0.43 (-0.76; -0.11)
Males	-0.03 (-0.06; -0.01)	-0.22 (-0.40; -0.04)	-0.29 (-0.62; 0.03)	0.18 (-0.26; 0.63)
Pulse pressure; mmHg				
Females	-0.15 (-0.17; -0.12)	-0.57 (-0.77; -0.38)	-0.94 (-1.26; -0.63)	-1.35 (-1.71; -0.98)
Males	-0.12 (-0.15; -0.09)	-0.21 (-0.41; 0.00)	-0.89 (-1.25; -0.52)	-1.04 (-1.53; -0.54)

	OR (95%-CI)	OR (95%-CI)	OR (95%-CI)	OR (95%-CI)
Hypertension				
Females	0.98 (0.97; 0.99)	0.91 (0.87; 0.96)	0.87 (0.80; 0.94)	0.87 (0.80; 0.94)
Males	0.99 (0.98; 0.99)	0.94 (0.90; 0.98)	0.90 (0.83; 0.97)	1.01 (0.91; 1.13)

Data are reported as β coefficients or odds ratios (OR) with 95% confidence intervals (CI) derived from linear or logistic regression models adjusted for age, years of formal education, body mass index, smoking status, intake of antihypertensive medication (not for outcome hypertension), and examination center

*Abbreviations: CM* Childhood Maltreatment, *CTS*_SUM_ Childhood Trauma Screener sum score

Excluding BMI and smoking from the adjustment set resulted in only minor attenuation of associations for continuous blood pressure outcomes (Table S2). Inverse associations with systolic blood pressure remained largely consistent, although estimates were slightly weakened and no longer statistically significant in highly exposed males. There was no clear evidence of an association with diastolic blood pressure irrespective of adjustment, while results for pulse pressure were virtually unchanged. In contrast, associations with hypertension were attenuated, with effect estimates shifting towards the null and losing statistical significance across most exposure categories.

### Sex-specific associations of cumulative CM exposure with SBP, DBP, PP, and hypertension

Sex-disaggregated results of cumulative CM-exposure (i.e., the experience of none, one, two, three or more moderate/severe CM subtypes) with BP markers showed an inverse dose-response relationship between CM and SBP, DBP, and PP in females (Table 2). Compared to females without CM-exposure, those who experienced one type of CM had a 0.69 mmHg lower SBP and 0.12 mmHg lower DBP. Females, who experienced three or more types of CM had on average a 1.78 mmHg lower SBP and 0.43 mmHg lower DBP compared to females without CM. The OR for hypertension was 0.87 (95% CI = 0.80; 0.94) in females, who had experienced ≥ 3 types of CM. In males, the overall association pattern between cumulative CM exposure and BP markers was also inverse without evidence of a dose-response relationship. In males with high cumulative CM exposure (≥ 3 types of CM), there was no elevated risk of hypertension (OR: 1.01, 95% CI: 0.91; 1.13). For sensitivity analyses, we repeated the analysis and additionally adjusted for migration status (Table S3), which revealed only minimal differences compared to the main findings, but did not change the overall pattern of associations.

### Age- and sex-specific associations of CTS_SUM_ with SBP, DBP and PP

**Fig. 1:**
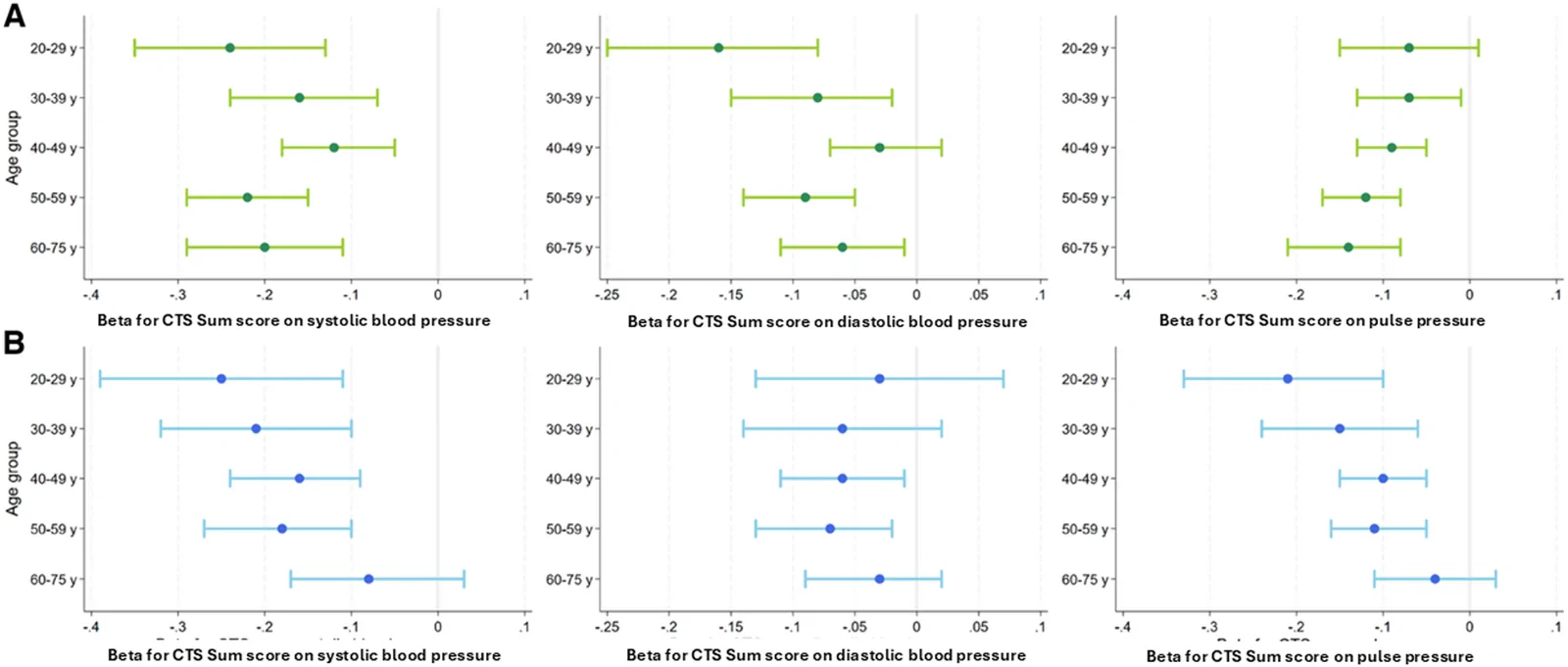
Age-stratified associations of the CTS_SUM_ with blood pressure markers in females (green color) and males (blue color). The upper panel (**A**) displays results for females (green color), the lower panel (**B**) for males (blue color). The figures on the left illustrate the association with SBP, those in the center with DBP, and those on the right with PP. Data are presented by age groups: 20–29, 30–39, 40–49, 50–59, and 60 + years. β-coefficients represent the change per one-point increase in CTS sum score. Note: Data are reported as β-coefficients with 95% confidence intervals (95% CI) derived from age- and sex-stratified linear regression models adjusted for age (within age-groups), education, body mass index, smoking status, antihypertensive medication use, and study center. Abbreviations: SBP = systolic blood pressure; DBP = diastolic blood pressure; PP = pulse pressure; CTS = Childhood Trauma Screener; CTSSUM = Childhood Trauma Screener sum score

Across different age groups sex-specific associations between CM and BP markers are shown in Table 3; Fig. 1. Age-CTS-interactions (Table S4) indicate that age is an effect modifier for all CTS-BP-associations (all *p*≤0.001). Across age groups and sexes, inverse associations between CM and SBP with varying effect sizes were observed, representing cross-sectional differences between age groups. In females, the inverse association between CTS_SUM_ and SBP was strongest in the youngest age group, weakened progressively up to the age of 50 years, and then became stronger again in older age groups. The inverse association between CTS_SUM_ and DBP was also strongest in the youngest females, but decreased with age and was weakest in females aged 40–49 years. In contrast, the inverse association between CTS_SUM_ and PP became progressively stronger with age in females.

**Table 3:** Age group and sex-specific associations of the CTS_SUM_ with blood pressure markers

	Age group									
	20–29 years			30–39 years			40–49 years	50–59 years	60 + years	
Systolic blood pressure										
Females	-0.24 (-0.35; -0.13)			-0.16 (-0.24; -0.07)			-0.12 (-0.18; -0.05)	-0.22 (-0.29; -0.15)	-0.20 (-0.29; -0.11)	
Males	-0.25 (-0.39; -0.11)			-0.21 (-0.32; -0.10)			-0.16 (-0.24; -0.09)	-0.18 (-0.27; -0.10)	-0.08 (-0.17; 0.03)	
Diastolic blood pressure										
Females	-0.16 (-0.25; -0.08)			-0.08 (-0.15; -0.02)			-0.03 (-0.07; 0.02)	-0.09 (-0.14; -0.05)	-0.06 (-0.11; -0.01)	
Males	-0.03 (-0.13; 0.07)			-0.06 (-0.14; 0.02)			-0.06 (-0.11; -0.01)	-0.07 (-0.13; -0.02)	-0.03 (-0.09; 0.02)	
Pulse pressure										
Females	-0.07 (-0.15; 0.01)			-0.07 (-0.13; -0.01)			-0.09 (-0.13; -0.05)	-0.12 (-0.17; -0.08)	-0.14 (-0.21; -0.08)	
Males	-0.21 (-0.33; -0.10)			-0.15 (-0.24; -0.06)			-0.10 (-0.15; -0.05)	-0.11 (-0.16; -0.05)	-0.04 (-0.11; 0.03)	

Data are reported as β-coefficients with 95% confidence intervals (95% CI) derived from age- and sex-stratified linear regression models adjusted for age (within age-groups), education, body mass index, smoking status, antihypertensive medication use, and study center

*Abbreviations: CTS*_SUM_ Childhood Trauma Screener sum score

In males, the inverse association between CTS_SUM_ and SBP weakened with increasing age and diminished in the 60 + group. The strongest inverse associations between CTS_SUM_ and DBP were observed in males aged 30–59 years. Associations between CTS_SUM_ and PP decreased steadily with age in males. These results are presented in Figure S4 in mmHg. For sensitivity analyses, we repeated the analysis excluding all participants taking antihypertensive medication (Table S5), which revealed only minimal differences compared with the main findings. Most associations were slightly stronger after exclusion, with an average additional reduction of 0.01 mmHg in BP per one-point increase in CTS_SUM_.

Across age groups, exclusion of BMI and smoking resulted in only minor changes in the associations for continuous blood pressure outcomes (Table S6). For systolic blood pressure, estimates were slightly attenuated in some age groups, particularly among younger females, but the overall pattern of lower systolic blood pressure with higher maltreatment exposure remained consistent across ages. There was no clear evidence of an association with diastolic blood pressure in either model, with small effect estimates and confidence intervals frequently including the null across all age groups. Results for pulse pressure were largely unchanged, with similar effect sizes and consistent patterns across age strata, indicating minimal influence of BMI and smoking on these associations.

### Age- and sex-specific associations of CTS subscales with SBP, DBP and PP

We examined sex-specific associations of different CTS subscales with BP markers (Table S7). Overall, the associations of the CTS subscales with SBP and PP were more pronounced than those with DBP in both sexes and were predominantly inverse. The sex-specific associations of the different CTS subscales with SBP, DBP, and PP across different age groups are presented in Table S8. The age-specific inverse associations were more consistent for the abuse subscales than for the neglect subscales, and were more pronounced in females.

## Discussion

We analyzed the age- and sex-dependent associations between CM exposure and BP-based outcomes in a large population-based cross-sectional study. We observed significant age- and sex-dependent associations between CM and BP markers, indicating between-group differences across age strata. CM exposure was inversely associated with SBP across all age groups except for male participants aged 60 + years. For DBP, the inverse association with CM was most pronounced in young females and weakened with age, while in males, it was only pronounced in middle-aged participants. Only in females and for both SBP and DBP, we observed a U-shaped trend with decreasing CM- BP associations until mid-age (40–49 years), followed by an increase from age 50 years. CM-PP-associations exhibited opposing sex- and age-dependent trends: While in females, the inverse associations became stronger with increasing age, the inverse associations in males became weaker with increasing age.

Overall, associations appeared more consistent in females in sex-stratified analyses, with formal evidence of sex interaction for systolic blood pressure and pulse pressure, but not for diastolic blood pressure or hypertension. Moreover, inverse associations were observed more consistently for abuse than for neglect items in both females and males across age groups.

Given that CM is associated with increased CVD risk [6, 11], the general pattern of inverse associations between CM and BP markers may initially appear counterintuitive. For instance, the recent meta-analysis by Niu et al. [11] demonstrated that CM is associated with 19% higher odds of hypertension. In the same analysis, no evidence for an association of CM exposure with SBP and only a weak effect (Cohen´s d = 0.04) on DBP was observed. Moderator analyses did not provide evidence of effect modification by either age or sex on any outcome. However, our results are partly consistent with previous studies that also demonstrated negative associations [14–16]. For example, Terock et al. [16], which was not considered in the meta-analysis by Niu et al. [11] observed lower SBP and DBP in individuals with CM exposure in a cross-sectional analysis in large population-based sample, and Winzeler et al. [17] showed a decrease in SBP with CM exposure among young females, paralleling our findings. In a longitudinal study with repeated BP measurements, Souama et al. reported a significant interaction between CM and time, with severe CM being associated with a lower SBP at initial assessment and higher SBP at 9-years follow-up compared to individuals without CM. They did not observe a CM-time interaction for DBP [14]. Importantly, Souama et al. controlled for sex and age in their analytical models, whereas we report sex- and age-disaggregated data. Furthermore, they calculated mean BP values based on the first two consecutive readings, which were assessed in the supine position, while BP outcomes in our study are solely based on the second measurement, which was obtained in the sitting position [22]. Notably, SBP tends to be approximately 5 mmHg higher in the supine position than in the sitting position [24]. The European Society of Cardiology [23] currently recommends measuring BP in the sitting position as the standard approach. These methodological differences raise the question of whether individuals with a history of CM might exhibit an overlooked form of isolated supine hypertension without seated hypertension, a distinct pattern of BP dysregulation that has recently attracted research interest in the context of CVD risk [25].

Another longitudinal study with a 23-year follow-up in a small (*n* = 394), ethnically diverse sample (ages between 5 and 16 years at baseline), reported an interaction between CM and age, showing a more rapid increase in SBP and DBP after age 30 in association with CM [13]. While this longitudinal analysis provides important data on the CM- BP association, their sample was significantly younger and smaller than the NAKO sample. It should be emphasized that both longitudinal studies [13, 14] model trajectories of CM-associated alterations in cardio-metabolic health [14] and BP development over time [13], whereas we estimated sex-and age-dependent CM-BP associations cross-sectionally. Overall, this between-study heterogeneity limits comparability, and we propose that our approach of sex- and age-disaggregated analyses may contribute valuable additional insights into the intricate association between CM and BP-related outcomes. Although BP was measured at rest in the NAKO, it may also be worthwhile to consider data that assess CM-associated cardiovascular reactivity to acute stressors. A meta-analysis of 83 studies concluded that CM and other forms of adverse childhood experiences (e.g., household dysfunction) were associated with blunted cardiovascular stress reactivity [26], especially in younger individuals irrespective of sex, and in individuals with greater exposure severity. These data may suggest a generalized downregulation of BP both at baseline and after acute stress exposure in individuals with CM-exposure.

Indeed, numerous attempts have been made to link CM exposure to alterations in adult cardiovascular stress-physiology [27, 28], which may provide a plausible mechanistic context for our findings of lower resting BP in individuals with CM exposure. The nervous, endocrine, and immune systems are highly integrated and maintain physiological equilibrium under stress through adaptive responses, a mechanism termed *allostasis* [29]. Under stress, the locus-coeruleus noradrenergic network is especially relevant and has been shown to be functionally and structurally altered in individuals exposed to CM [30]. Evidence further suggests that other components of the allostatic system, including the neuroendocrine renin-angiotensin-aldosterone system (RAAS), the hypothalamic–pituitary–adrenal (HPA) axis and the immune system, as well as behavioral factors and epigenetic modifications in stress-regulatory pathways, may be particularly important in mediating long-term BP alterations in individuals exposed to CM [27, 28]. For example, the RAAS, which is crucial for BP regulation [31], shows distinct patterns in individuals with CM including increased aldosterone without changes in renin levels [16]. These associations have been observed for abuse but not for neglect, which fits well with our finding of more consistent associations for abuse-related CM. CM is also linked to hypo reactivity of the HPA axis, as reflected by blunted plasma cortisol after acute stress, especially in females [32]. It has been suggested that CM-associated long-term HPA-axis hyporeactivity via glucocorticoid receptor resistance may contribute to baroreceptor desensitization through multiple pathways [33]. While desensitization of cardiovascular stress-response systems may be adaptive in the short term, long-term dysregulation may increase the risk of CVD. Despite remarkable advances in our understanding of how CM may contribute to increased CVD risk in later life, there is still a substantial knowledge gap regarding the roles of sex and gender in this context. At the physiological level, studies in humans and rodents have provided compelling evidence of sex differences in BP regulation [34], but few studies have addressed sex-related modifications of the association between CM exposure and adult cardiovascular risk.

Of note, our data reveal decreased association strengths in female participants around age 50, which may coincide with the menopausal transition [35], raising important questions that are specific to female individuals. Given that basic research on autonomic control of BP regulation has historically neglected females, very little is known about the effect of estrogen withdrawal on the autonomic response to stress [34] and, to our knowledge, nothing is known about the influence of perimenopausal hormonal changes in research on long-term cardiovascular outcomes associated with CM.

Several limitations of this study should be noted. First, the analysis is based on cross-sectional data from the NAKO baseline assessment, preventing conclusions about individual developmental changes in the relationship between CM and BP across the lifespan. Observed age differences likely reflect cohort effects but not changes over time, an important limitation which should be disentangled using longitudinal data. As an observational study, causality cannot be inferred. The NAKO baseline response of 17% [19] raises the possibility of selection bias, as participants may differ systematically from non-respondents in terms of health or CM exposure. In particular, selection processes related to mental or physical health, as well as mortality, may have contributed to an underrepresentation of individuals with CM who experience more severe health consequences and therefore may have a tendency towards higher BP levels. Of the 204,744 participants in the NAKO baseline assessment, 53,761 were excluded from the present analysis due to missing data, the majority of whom (*n* = 32,772) were excluded due to missing CTS data. It is possible that individuals affected by CM were less likely to complete the CTS, potentially introducing selection bias through selective participation or responding. Among those who completed the CTS, underreporting and reporting bias cannot be excluded. Retrospective assessment of CM is particularly susceptible to recall bias and non-disclosure, which may lead to an underestimation of CM exposure and, consequently, bias the observed associations. In addition, CM was assessed retrospectively using a single item from the CTQ for each CM dimension, selected for its high discriminative power [20]. Although this approach improves feasibility in large cohorts, it may reduce measurement precision and limit sensitivity compared to the full CTQ questionnaire. Next, and given the number of outcomes and stratified analyses, the potential for type I error due to multiple testing should be considered. Our analyses were guided by a priori hypotheses on the association between childhood maltreatment and blood pressure, including effect modification by age and sex, whereas subtype-specific analyses were more exploratory. We did not apply formal multiplicity correction, as our focus was on the overall pattern and consistency of associations rather than isolated significant findings. Nevertheless, results from exploratory analyses should be interpreted with caution. Moreover, NAKO participants provided self-reports on a dichotomous question (“female”, “male”) about their sex. However, the German language does not distinguish between sex and gender (“Geschlecht”), which may contribute to a conflation or inconsistent use of sex and gender concepts in the German research context. Further questions about e.g., gender identity and gender roles, were not asked and represent an important gap that should be addressed in future research, given the widely acknowledged roles of both sex and gender as modifiers of health and disease [36]. Finally, BMI was used as an indicator of obesity. We acknowledge that BMI may only inaccurately capture obesity because of its inability to differentiate between fat and muscle mass.

Strengths of this study include the use of standardized, on-site BP measurements in accordance with current guidelines, which provide more objective data and may partly mitigate aforementioned problems associated with recall and measurement bias. As demonstrated by Reuben et al. [37], retrospective assessments of adverse childhood experiences tend to show stronger associations with subjective outcomes than with objectively recorded health data, making the use of objectively measured BP particularly valuable. However, as BP was assessed at a single visit, there may be potential inaccuracy in the measurements, particularly compared with repeated office BP measurements over time or 24-hour ambulatory BP monitoring, which represent standard approaches in routine clinical practice [23]. Although statistical models were adjusted for antihypertensive medication use and sensitivity analyses excluding treated participants yielded results consistent with the main findings, BP interpretation in participants receiving antihypertensive treatment remains limited, as antihypertensive therapy may introduce treatment-related or collider bias. A further strength of this study is the large sample size, which improves statistical power and allows for more reliable estimates of associations, including in individuals with high cumulative CM exposure. Finally, the age and sex-disaggregated data analyses are in agreement with current efforts to systematically integrate intersectional analyses into research [38].

There is strong evidence that CM is associated with an increased risk of CVD [6]. Given that BP plays an essential role in cardiovascular health and life expectancy, elevated BP is a key parameter in risk assessment and preventive care for determining CVD risk [39]. However, if individuals with a history of CM are at increased CVD risk, while not necessarily exhibiting higher BP levels or even showing lower odds of hypertension, they may not be adequately identified through standard CVD screening programs. Therefore, healthcare professionals should be aware that this vulnerable group may remain at increased cardiovascular risk despite resting BP levels that are comparable to or even lower than those of the general population, suggesting that BP-based screening alone may not fully capture their cardiovascular risk. Future research identifying the CM-related risk factors that most strongly contribute to CVD risk would be of high value in guiding clinical prevention and care, ultimately supporting global efforts to improve population health. While the observed associations between CM and BP values are small in magnitude and may have limited clinical consequences at the individual level in terms of acute cardiovascular health, they may nonetheless point towards subtle alterations in the underlying cardiovascular regulatory mechanisms in the context of CM exposure. In light of established evidence on the association between CM and CVD risk, these effects are more likely to be relevant at the population level than as determinants of individual hypertension risk.

The health risks associated with various BP levels also differ between sexes, with the increased risk of CVD associated with elevated BP begins at lower SBP in women compared to men. Given that at an SBP of 110–119 mmHg, women face a cardiovascular risk comparable to that of men with SBP levels of 140–149 mmHg [40], an important direction for future research is to explore the sex-specific clinical relevance of the findings from this study in more detail, such as investigating hypertension at a sex-specific SBP of ≥ 110 mmHg for women. To ensure more inclusive and effective healthcare strategies for all affected populations, as well as to gain a deeper understanding of the underlying mechanisms, future research should prioritize longitudinal analyses to investigate the long-term development of BP in relation to CM, along with a systematic investigation of sex, gender, age, and further diversity characteristics towards more personalized approaches in cardiovascular risk assessment and prevention.

The observed associations between CM and adult BP exhibit pronounced age- and sex-dependent variability, with stronger and more consistent associations observed in females, as well as differences across age groups in adulthood. Using BP parameters as an exemplar, our findings emphasize the complexity of the long-term physical consequences associated with CM, and highlight the importance of conducting sex- and age-specific analyses to gain a more nuanced understanding of these relationships.

## Supplementary Information

Supplementary Material 1.

## Data Availability

Access to and use of NAKO data and bio samples can be obtained via an electronic application portal (https://transfer.nako.de).

## Abbreviations

ATC: Anatomical therapeutic chemical codes
BMI: Body mass index
BP: Blood pressure
CI: Confidence interval
CM: Child maltreatment
CTS: Childhood Trauma Screener
CTQ: Childhood Trauma Questionnaire
CVD: Cardiovascular disease
DBP: Diastolic blood pressure
HPA: Hypothalamic–pituitary–adrenal axis
NAKO: German National Cohort (Nationale Kohorte)
OR: Odds ratio
PP: Pulse pressure
RAAS: Renin-angiotensin-aldosterone system
SBP: Systolic blood pressure
