# Supplementary material for: Childhood maltreatment and adult blood pressure-based outcomes: age- and sex-specific associations in the German National Cohort (NAKO)
Source: BMC Public Health. 2026 Sep 9;26:2698. doi: 10.1186/s12889-026-29019-z (PMC13621648; doi:10.1186/s12889-026-29019-z)
Supplement: Supplementary file 1 [file 12889_2026_29019_MOESM1_ESM.docx]

**Supplement**

Childhood maltreatment and adult blood pressure-based outcomes: age- and sex-specific associations in the German National Cohort (NAKO)

**Figure S1:** Study flow chart of sequential exclusion of participants with missing data for main variables**.**

**Supplemental Figure S2.** Age distribution of systolic blood pressure (A), diastolic blood pressure (B), and pulse pressure (C) for female and male participants**.**

**Figure S3.** Age distribution of mean CTS sum score for female and male participants.

**Abbreviation:** CTS = Childhood Trauma Screener.

**Table S1**. p-values for the interaction of sex and the CTS sum score on blood pressure measurements

| **Marker** | **p** |
| --- | --- |
| **Systolic blood pressure; mmHg** | <0.001 |
| **Diastolic blood pressure; mmHg** | 0.327 |
| **Pulse pressure; mmHg** | <0.001 |
| **Hypertension** | 0.706 |

**Note:** Results are expressed as p values for the interaction of sex with childhood maltreatment (CTS sum score) on blood pressure measurements derived from regression models adjusted for education, body mass index, smoking status, examination center, and antihypertensive medication (only for continuous measurements)

**Table S2.** Sex-specific associations of childhood maltreatment (CM) and cumulative CM with blood pressure markers (reduced adjustment)

| Outcome | **CTS_SUM_**  **β (95%-CI)** | **1 vs. no CM**  **β (95%-CI)** | **2 vs. no CM**  **β (95%-CI)** | **3+ vs. no CM**  **β (95%-CI)** |
| --- | --- | --- | --- | --- |
| **Systolic blood pressure; mmHg**  Females  Males | -0.18 (-0.22; -0.14)  -0.13 (-0.18; -0.09) | -0.66 (-0.94; -0.38)  -0.35 (-0.64; -0.07) | -1.23 (-1.69; -0.77)  -1.06 (-1.56; -0.57) | -1.47 (-2.00; -0.95)  -0.64 (-1.32; 0.03) |
| **Diastolic blood pressure; mmHg**  Females  Males | -0.02 (-0.05; 0.00)  -0.14 (-0.33; 0.05) | -0.04 (-0.22; 0.14)  -0.14 (-0.33; 0.05) | -0.22 (-0.51; 0.08)  -0.14 (-0.47; 0.19) | -0.05 (-0.38; 0.18)  0.44 (-0.00; 0.89) |
| **Pulse pressure; mmHg**  Females  Males | -0.16 (-0.18; -0.13)  -0.12 (-0.16; -0.09) | -0.62 (-0.81; -0.43)  -0.22 (-0.42; -0.01) | -1.02 (-1.33; -0.70)  -0.92 (-1.29; -0.56) | -1.42 (-1.79; -1.06)  -1.09 (-1.58; -0.59) |
|  |  | **OR (95%-CI)** | **OR (95%-CI)** | **OR (95%-CI)** |
| **Hypertension**  Females  Males | 0.99 (0.98; 0.99)  1.00 (0.99; 1.00) | 0.94 (0.90; 0.98)  0.97 (0.93; 1.01) | 0.95 (0.88; 1.02)  0.95 (0.88; 1.03) | 1.01 (0.93; 1.09)  1.10 (0.99; 1.21) |

**Note:** Data are reported as β coefficients or odds ratios (OR) with 95% confidence intervals (CI) derived from linear or logistic regression models adjusted for age, years of formal education, intake of antihypertensive medication (not for outcome hypertension), and examination center.

**Abbreviations:** CM = Childhood Maltreatment; CTS_SUM_ = Childhood Trauma Screener sum score.

**Table S3.** Sex-specific associations of childhood maltreatment (CM) and cumulative CM with blood pressure markers (additionally adjusted for migration status)

| **Outcome** | **CTS_SUM_**  **β (95%-CI)** | **1 vs. no CM**  **β (95%-CI)** | **2 vs. no CM**  **β (95%-CI)** | **3+ vs. no CM**  **β (95%-CI)** |
| --- | --- | --- | --- | --- |
| **Systolic blood pressure; mmHg**  Females  Males | -0.19 (-0.23; -0.15)  -0.14 (-0.18; -0.10) | -0.64 (-0.92; -0.36)  -0.37 (-0.65; -0.09) | -1.30 (-1.76; -0.84)  -1.05 (-1.54; -0.55) | -1.70 (-2.23; -1.18)  -0.77 (-1.44; -0.10) |
| **Diastolic blood pressure; mmHg**  Females  Males | -0.05 (-0.07; -0.02)  -0.03 (-0.06; 0.00) | -0.10 (-0.27; 0.08)  -0.20 (-0.39; -0.02) | -0.40 (-0.69; -0.11)  -0.26 (-0.58; 0.07) | -0.41 (-0.74; -0.08)  0.21 (-0.24; 0.65) |
| **Pulse pressure; mmHg**  Females  Males | -0.14 (-0.17; -0.11)  -0.11 (-0.14; -0.08) | -0.54 (-0.73; -0.34)  -0.17 (-0.37; 0.04) | -0.90 (-1.22; -0.58)  -0.79 (-1.15; -0.42) | -1.30 (-1.66; -0.93)  -0.97 (-1.47; -0.48) |
|  | **OR (95%-CI)** | **OR (95%-CI)** | **OR (95%-CI)** | **OR (95%-CI)** |
| **Hypertension**  Females  Males | 0.98 (0.97; 0.99)  0.99 (0.98; 1.00) | 0.92 (0.87; 0.96)  0.95 (0.91; 0.99) | 0.87 (0.80; 0.94)  0.91 (0.85; 0.99) | 0.87 (0.80; 0.95)  1.02 (0.92; 1.14) |

**Note:** Data are reported as β coefficients or odds ratios (OR) with 95% confidence intervals (CI) derived from linear or logistic regression models adjusted for age, years of formal education, body mass index, smoking status, intake of antihypertensive medication (not for outcome hypertension), examination center, and migration status.

**Abbreviations:** CM = Childhood Maltreatment; CTS_SUM_ = Childhood Trauma Screener sum score.

**Table S4**. p-values for the interaction of age and the CTS sum score on blood pressure measurements

| **Marker** | **Females** | **Males** |
| --- | --- | --- |
| **Systolic blood pressure; mmHg** | 0.001 | <0.001 |
| **Diastolic blood pressure; mmHg** | <0.001 | <0.001 |
| **Pulse pressure; mmHg** | <0.001 | <0.001 |
| **Hypertension** | 0.115 | 0.001 |

Results are expressed as p values for the interaction of age with childhood maltreatment (CTS sum score) on blood pressure measurements derived from regression models adjusted for education, body mass index, smoking status, examination center, and antihypertensive medication (only for continuous measurements)

**Supplemental Figure S4.** Age group and sex-specific associations of the CTS_SUM_ with blood pressure markers

The upper panel displays results for women, the lower panel for men. The figures on the left illustrate the association with SBP, those in the center with DBP, and those on the right with PP. Data are presented by age groups: 20–29 (blue colour), 30–39 (orange colour), 40–49 (green colour), 50–59 (yellow colour), and 60+ (red colour) years. Blood pressure is shown in mmHg. **Note:** Data are reported as β-coefficients with 95% confidence intervals (95% CI) derived from age- and sex-stratified linear regression models adjusted for age (within age-groups), education, body mass index, smoking status, antihypertensive medication use, and study center.

**Abbreviations:** SBP = systolic blood pressure; DBP = diastolic blood pressure; PP = pulse pressure; CTS = Childhood Trauma Screener; CTS_SUM_ = Childhood Trauma Screener sum score.

**Supplemental Table S5.** BP medication excluded age group and sex-specific associations of the CTS sum score with blood pressure markers

|  |  |  |  | |  | **Age group** | | | |  |
| --- | --- | --- | --- | --- | --- | --- | --- | --- | --- | --- |
|  | 20 - 29 | | | 30 - 39 | | | 40 - 49 | 50 – 59 | 60+ | |
| **Systolic blood pressure**  Females  Males | -0.24 (-0.34; -0.13)  -0.27 (-0.41; -0.13) | | | -0.16 (-0.25; -0.07)  -0.24 (-0.35; -0.12) | | | -0.12 (-0.19; -0.05)  -0.18 (-0.26; -0.10) | -0.23 (-0.31; -0.15)  -0.20 (-0.30; -0.10) | -0.32 (-0.44; -0.20)  -0.01 (-0.15; 0.12) | |
| **Diastolic blood pressure**  Females  Males | -0.17 (-0.25; -0.08)  -0.04 (-0.14; 0.06) | | | -0.09 (-0.16; -0.02)  -0.06 (-0.14; 0.03) | | | -0.04 (-0.09; 0.01)  -0.08 (-0.14; -0.03) | -0.10 (-0.15; -0.05)  -0.10 (-0.17; -0.04) | -0.10 (-0.16; -0.03)  -0.06 (-0.14; 0.02) | |
| **Pulse pressure**  Females  Males | -0.07 (-0.15; 0.01)  -0.22 (-0.34; -0.10) | | | -0.07 (-0.13; -0.01)  -0.18 (-0.27; -0.10) | | | -0.08 (-0.12; -0.04)  -0.10 (-0.15; -0.04) | -0.13 (-0.18; -0.08)  -0.09 (-0.16; -0.03) | -0.22 (-0.31; -0.14)  0.05 (-0.05; 0.15) | |

**Note:** Data are reported as β-coefficients with 95% confidence intervals (95% CI) derived from age- and sex-stratified linear regression models adjusted for age (within age-groups), education, body mass index, smoking status, and study center.

**Table S6.** Age group and sex-specific associations of the CTS_SUM_ with blood pressure markers (reduced adjustment)

|  |  |  |  | |  | **Age group** | | | |  |
| --- | --- | --- | --- | --- | --- | --- | --- | --- | --- | --- |
|  | 20 – 29 years | | | 30 – 39 years | | | 40 – 49 years | 50 – 59 years | 60+ years | |
| **Systolic blood pressure**  Females  Males | -0.16 (-0.27; -0.04)  -0.22 (-0.36; -0.08) | | | -0.13 (-0.22; -0.03)  -0.17 (-0.28; -0.06) | | | -0.07 (-0.13; 0.01)  -0.14 (-0.22; -0.07) | -0.21 (-0.28; -0.14)  -0.17 (-0.25; -0.09) | -0.22 (-0.31; -0.13)  -0.07 (-0.17; 0.03) | |
| **Diastolic blood pressure**  Females  Males | -0.11 (-0.20; -0.02)  -0.01 (-0.11; 0.09) | | | -0.06 (-0.14; 0.01)  -0.02 (-0.11; 0.06) | | | 0.02 (-0.03; 0.06)  -0.03 (-0.09; 0.02) | -0.08 (-0.12; -0.03)  -0.06 (-0.11; -0.01) | -0.06 (-0.11; -0.01)  -0.04 (-0.09; 0.02) | |
| **Pulse pressure**  Females  Males | -0.05 (-0.13; 0.03)  -0.21 (-0.32; -0.09) | | | -0.06 (-0.12; -0.01)  -0.15 (-0.23; -0.06) | | | -0.08 (-0.13; -0.04)  -0.11 (-0.16; -0.06) | -0.13 (-0.18; -0.08)  -0.11 (-0.17; -0.05) | -0.16 (-0.22; -0.09)  -0.03 (-0.11; 0.04) | |

**Note:** Data are reported as β-coefficients with 95% confidence intervals (95% CI) derived from age- and sex-stratified linear regression models adjusted for age (within age-groups), education, antihypertensive medication use, and study center.

**Abbreviations:** CTS_SUM_ = Childhood Trauma Screener sum score.

**Supplemental Table S7.** Sex-specific associations of specific forms of childhood maltreatment with blood pressure markers

| **Outcome** | **Physical neglect** | **Emotional neglect** | **Physical abuse** | **Emotional abuse** | **Sexual abuse** |
| --- | --- | --- | --- | --- | --- |
|  | **β (95%-CI)** | **β (95%-CI)** | **β (95%-CI)** | **β (95%-CI)** | **β (95%-CI)** |
| **Systolic blood pressure; mmHg**  Females  Males | -0.02 (-0.37; 0.34)  0.31 (-0.03; 0.66) | -1.55 (-1.93; -1.17)  -1.16 (-1.58; -0.75) | -1.26 (-1.66; -0.86)  -0.95 (-1.32; -0.57) | -1.23 (-1.59; -0.88)  -0.86 (-1.28; -0.44) | -1.24 (-1.60; -0.88)  -1.29 (-1.94; -0.65) |
| **Diastolic blood pressure; mmHg**  Females  Males | -0.04 (-0.27; 0.18)  0.07 (-0.16; 0.30) | -0.28 (-0.53; -0.04)  -0.07 (-0.33; 0.21) | -0.37 (-0.62; -0.12)  -0.22 (-0.46; 0.03) | -0.37 (-0.59; -0.15)  -0.24 (-0.52; 0.04) | -0.28 (-0.50; -0.05)  -0.19 (-0.61; 0.24) |
| **Pulse pressure; mmHg**  Females  Males | 0.03 (-0.22; 0.28)  0.25 (-0.01; 0.51) | -1.27 (-1.54; -1.00)  -1.10 (-1.41; -0.79) | -0.89 (-1.17; -0.61)  -0.72 (-1.00; -0.45) | -0.86 (-1.11; -0.61)  -0.61 (-0.93; 0.30) | -0.96 (-1.22; -0.71)  -1.11 (-1.59; -0.63) |
|  | **OR (95%-CI)** | **OR (95%-CI)** | **OR (95%-CI)** | **OR (95%-CI)** | **OR (95%-CI)** |
| **Hypertension**  Females  Males | 0.99 (0.94; 1.05)  1.00 (0.95; 1.05) | 0.88 (0.83; 0.94)  0.94 (0.88; 1.00) | 0.89 (0.83; 0.95)  0.94 (0.88; 1.00) | 0.93 (0.88; 0.99)  0.96 (0.90; 1.03) | 0.86 (0.81; 0.92)  0.88 (0.79; 0.97) |

Data are reported as β coefficients or odds ratios (OR) with 95% confidence intervals (CI) derived from linear or logistic regression models adjusted for age, years of schooling, body mass index, smoking status, intake of antihypertensive medication (not for outcome hypertension), and examination center.

**Supplemental Table S8.** Age group and sex-specific associations of CTS subscales with blood pressure markers

|  | **Age group** | | | | |
| --- | --- | --- | --- | --- | --- |
| **Physical neglect** | 20 – 29 years | 30 – 39 years | 40 – 49 years | 50 – 59 years | 60+ years |
| **Systolic blood pressure**  Females  Males | -1.44 (-2.73; -0.15)  -1.08 (-2.57; 0.41) | 0.52 (-0.45; 1.49)  -0.06 (-1.12; 1.00) | -0.33 (-1.01; 0.35)  0.13 (-0.50; 0.75) | -0.65 (-1.32; 0.03)  0.20 (-0.46; 0.85) | 0.61 (-0.14; 1.35)  0.62 (-0.08; 1.31) |
| **Diastolic blood pressure**  Females  Males | -1.03 (-2.05; 0.00)  -0.15 (-1.21; 0.91) | 0.44 (-0.32; 1.19)  0.14 (-0.63; 0.90) | 0.18 (-0.29; 0.64)  0.36 (-0.08; 0.81) | -0.44 (-0.85; -0.04)  0.16 (-0.26; 0.58) | 0.21 (-0.19; 0.61)  0.09 (-0.30; 0.49) |
| **Pulse pressure**  Females  Males | -0.41 (-1.36; 0.53)  -0.94 (-2.18; 0.31) | 0.08 (-0.57; 0.73)  -0.19 (-1.00; 0.61) | -0.51 (-0.93; -0.08)  -0.24 (-0.67; 0.19) | -0.20 (-0.65; 0.24)  0.04 (-0.41; 0.48) | 0.40 (-0.15; 0.95)  0.52 (0.01; 1.04) |
| **Emotional neglect** |  | | | | |
| **Systolic blood pressure**  Females  Males | -2.81 (-4.28; -1.34)  -1.06 (-2.67; 0.55) | -0.24 (-1.27; 0.80)  -0.71 (-1.92; 0.50) | -0.80 (-1.48; -0.10)  -1.33 (-2.06; -0.60) | -1.68 (-2.39; -0.98)  -1.78 (-2.54; -1.03) | -1.87 (-2.73; -1.02)  -0.46 (-1.35; 0.44) |
| **Diastolic blood pressure**  Females  Males | -2.04 (-3.20; -0.87)  0.86 (-0.29; 2.00) | 0.26 (-0.55; 1.07)  0.038 (-0.84; 0.92) | -0.02 (-0.49; 0.45)  -0.31 (-0.84; 0.21) | -0.45 (-0.87; -0.02)  -0.54 (-1.03; -0.05) | -0.57 (-1.03; -0.11)  -0.03 (-0.53; 0.48) |
| **Pulse pressure**  Females  Males | -0.77 (-1.85; 0.30)  -1.92 (-3.26; -0.58) | -0.50 (-1.20; 0.20)  -0.75 (-1.67; 0.17) | -0.77 (-1.21; -0.34)  -1.02 (-1.52; -0.51) | -1.23 (-1.70; -0.77)  -1.24 (-1.76; -0.73) | -1.30 (-1.94; -0.67)  -0.43(-1.10; 0.23) |
| **Physical abuse** |  |  |  |  |  |
| **Systolic blood pressure**  Females  Males | -1.27 (-2.47; -0.10)  -1.53 (-2.89; -0.15) | -1.36 (-2.29; -0.42)  -1.27 (-2.33; -0.21) | -0.46 (-1.17; 0.26)  -1.00 (-1.67; -0.32) | -1.04 (-1.78; -0.29)  -1.07 (-1.75; -0.39) | -1.94 (-2.88; -1.00)  -0.63 (-1.42; 0.16) |
| **Diastolic blood pressure**  Females  Males | -1.11 (-2.05; -0.17)  -0.02 (-0.99; 0.96) | -0.73 (-1.46; 0.00)  -0.11 (-0.88; 0.66) | 0.02 (-0.47; 0.50)  -0.36 (-0.84; 0.12) | -0.39 (-0.84; 0.06)  -0.21 (-0.65; 0.22) | -0.78 (-1.29; -0.27)  -0.37 (-0.82; 0.08) |
| **Pulse pressure**  Females  Males | -0.18 (-1.04; 0.69)  -1.43 (-2.57; -0.29) | -0.63 (-1.26; 0.00)  -1.16 (-1.97; -0.35) | -0.47 (-0.92; -0.03)  -0.64 (-1.10; -0.74) | -0.65 (-1.14; -0.16)  -0.85 (-1.32; -0.39) | -1.16 (-1.86; -0.46)  -0.27 (-0.86; 0.32 |
| **Table S8 (continued). Age group and sex-specific associations of CTS subscales with blood pressure markers** | | | | | |
| **Emotional abuse** | 20 – 29 years | 30 – 39 years | 40 – 49 years | 50 – 59 years | 60+ years |
| **Systolic blood pressure**  Females  Males | -0.56 (-1.39; 0.27)  -1.52 (-2.63; -0.42) | -1.26 (-1.99; -0.54)  -0.75 (-1.73; 0.22) | -1.05 (-1.66; -0.45)  -1.43 (-2.13; -0.72) | -1.04 (-1.74; -0.35)  -0.83 (-1.66; 0.00) | -1.47 (-2.42; -0.52)  0.15 (-0.91; 1.21) |
| **Diastolic blood pressure**  Females  Males | -0.50 (-1.16; 0.16)  -0.51 (-1.30; 0.28) | -0.69 (-1.26; -0.12)  0.10 (-0.60; 0.81) | -0.31 (-0.72; 0.10)  -0.99 (-1.50; -0.49) | -0.53 (-0.95; -0.11)  -0.18 (-0.72; 0.35) | -0.45 (-0.97; 0.06)  -0.04 (-0.64; 0.56) |
| **Pulse pressure**  Females  Males | -0.06 (-0.66; 0.55)  -0.97 (-1.89; -0.04) | -0.58 (-1.07; -0.08)  -0.86 (-1.60; -0.12) | -0.75 (-1.16; -0.34)  -0.43 (-0.92; 0.05) | -0.52 (-0.98; -0.06)  -0.65 (-1.21; -0.85) | -1.02 (-1.72; -0.31)  0.19 (-0.59; 0.98) |
| **Sexual abuse** |  |  |  |  |  |
| **Systolic blood pressure**  Females  Males | -1.45 (-2.45; -0.46)  -0.68 (-2.74; 1.38) | -1.49 (-2.30; -0.69)  -1.39 (-2.95; 0.17) | -0.62 (-1.23; 0.00)  -2.48 (-3.52; -1.44) | -1.32 (-2.00; -0.63)  -1.11 (-2.34; 0.13) | -1.21 (-2.12; -0.31)  0.07 (-1.54; 1.67) |
| **Diastolic blood pressure**  Females  Males | -0.59 (-1.38; 0.21)  0.63 (-0.84; 2.09) | -0.68 (-1.31; -0.05)  -0.45 (-1.58; 0.69) | -0.28 (-0.70; 0.13)  -1.15 (-1.89; -0.41) | -0.60 (-1.01; -0.19)  -0.72 (-1.52; 0.07) | -0.22 (-0.71; 0.26)  0.51 (-0.40; 1.42) |
| **Pulse pressure**  Females  Males | -0.87 (-1.59; -0.14)  -1.31 (-3.03; 0.41) | -0.82 (-1.36; -0.27)  -0.95 (-2.13; 0.24) | -0.33 (-0.72; 0.06)  -1.33 (-2.04; -0.62) | -0.72 (-1.17; -0.27)  -0.39 (-1.23; 0.45) | -0.99 (-1.66; -0.33)  -0.44 (-1.63; 0.75) |

**Note:** Data are reported as β-coefficients with 95% confidence intervals (95% CI) derived from age- and sex-stratified linear regression models adjusted for age (within age-groups), education, body mass index, smoking status, antihypertensive medication use, and study center.

**Abbreviations:** CTS = Childhood Trauma Screener.
